# Integrated bulk and single-cell transcriptomic analyses with experimental validation identify PLOD1 as a diagnostic biomarker and TGF-β1-responsive profibrotic mediator in dupuytren’s disease

**DOI:** 10.3389/fmolb.2026.1924451

**Published:** 2026-09-30

**Authors:** Xianjie Lin, Mingming Wang, Yutian Lin, Yinshen Zhu, Zhijiang Ye, Haoran Chen

**Affiliations:** Department of Hand Surgery and Wound Repair Surgery, Wenzhou Medical College Affiliated Yueqing Hospital, Wenzhou, China

**Keywords:** dupuytren’s disease, FGF7, fibrosis, M2 macrophages, machine learning, PLOD1, single-cell RNA sequencing

## Abstract

**Background:**

Dupuytren’s disease (DD) is a progressive fibroproliferative disease characterized by palmar fascia fibrosis, extracellular matrix deposition, and finger contractures. Reliable diagnostic biomarkers and regulatory mechanisms have not yet been fully defined.

**Methods:**

Firstly, integrate the public transcriptome datasets GSE59746 and GSE75152. Then, limma was used to identify differentially expressed genes (DEGs) and combined with Weighted Gene Co expression Network Analysis (WGCNA) to screen candidate genes related to DD. PPI network analysis, functional enrichment, and four machine learning algorithms (LASSO, Boruta, Random Forest, and SVM-RFE) were applied to identify key biomarkers. Use CIBERSORT to assess immune infiltration. Analyze the single-cell RNA sequencing dataset GSE173252 to determine cell localization and pseudo time series analysis. Virtual knockout analysis is used to predict downstream regulatory targets. *In vitro* validation was conducted using TGF - β 1 stimulation and SB431542 inhibition.

**Results:**

A total of 598 DEGs were identified, and the WGCNA brown module showed the strongest correlation with DD. Cross analysis identified 346 candidate genes, mainly enriched in extracellular matrix tissue, collagen containing matrix, and collagen trimer formation. Procollagen-lysine, 2-oxoglutarate 5-dioxygenase 1 (PLOD1) has been identified as the only overlapping biomarker among the four machine learning algorithms. PLOD1 is significantly upregulated in DD tissues and exhibits strong diagnostic performance, with an AUC of 0.908. Immunoassay showed a decrease in M0 macrophage infiltration, while there was a positive correlation between PLOD1 and M2 macrophages. Single cell analysis showed that PLOD1 was mainly enriched in chondrocyte related populations and increased along the late pseudotime trajectory. Virtual knockout indicates that FGF7 is a key downstream gene. TGF - β 1 significantly induces PLOD1 and pro fibrotic markers, while SB431542 inhibits these effects.

**Conclusion:**

PLOD1 is a promising biomarker and potential functional regulator for DD diagnosis, closely related to extracellular matrix remodeling, pro fibrotic signaling, and immune microenvironment changes.

## Introduction

Dupuytren’s disease (DD) is a common progressive fibroproliferative disease that affects the palmar fascia of the hand. DD mainly affects the central area of the palmar aponeurosis and forms fibrous cords. These fibers are often attached to the base of the middle phalanx and can also be connected to the tendon sheath (Black and Blazar, 2011). The clinical feature of DD is that the contraction of the affected fascia will cause the fingers to bend irreversibly towards the palm, which will lead to serious loss of hand function and psychological incidence rate (Wilburn et al., 2013; Rehman et al., 2011). DD is a multifactorial disease, and genetics, alcohol, tobacco, and various systemic diseases may all contribute to the occurrence and development of DD (Michou et al., 2012; Wilkinson et al., 2012). The treatment of DD involves both surgical and non-surgical methods, all of which aim to eliminate the affected tissue (Thomas and Bayat, 2010; Bainbridge et al., 2012). Non surgical treatments mainly rely on radiation therapy, physical therapy, dimethyl sulfoxide solution, and injection of collagenase from *Clostridium difficile* (Sampson et al., 2011; Kaplan, 2011). However, the most effective treatment method is still to remove the fibrous cord that causes the patient’s symptoms through surgery (Thomas and Bayat, 2010; Bainbridge et al., 2012). However, even after effective surgery, patients may still experience permanent functional impairment due to established joint contractures, resulting in a high recurrence rate (van Rijssen et al., 2012). The risk of treatment failure and disease recurrence ranges from 8% to 66%. Therefore, it is necessary to further investigate the causes and related factors of this recurrence, and identify new diagnostic and therapeutic targets in order to improve the short-term and long-term prognosis of DD patients.

With the development of bioinformatics technology, bioinformatics analysis has become an indispensable part of biomedical research. Researchers can efficiently analyze the expression data of disease-related genes, gain a deeper understanding of the pathological and biological mechanisms of tumor or non tumor diseases from a genetic perspective, and reveal the key role of genes in disease development (Wang et al., 2024; Dong et al., 2025; Ubaid et al., 2025; Wu et al., 2023). A study has identified two genes (POSTN and COL11A1) as candidate diagnostic genes for DD and Frozen shoulder (FS) (Ouyang et al., 2023). Furthermore, machine learning is an emerging field with abundant resources to handle large, complex, and diverse data. It gradually improves our ability to search for relevant features in large high-dimensional data from gene expression profiles (Toh et al., 2019). Therefore, in this study, we systematically reviewed existing transcriptome analysis datasets and identified gene biomarkers associated with DD diagnosis through consensus using four different supervised machine learning feature selection techniques. Further explored the role of biomarkers in the pathogenesis of DD and potential candidates for therapeutic interventions.

## Methods

### Data source

The data used in this study (GSE59746, GSE75152, and GSE173252) were obtained from the Gene Expression Omnibus (GEO) database. As shown in Table 1, the GSE75152 dataset contains 12 control samples and 12 DD samples. The GSE59746 dataset includes 2 DD samples and 4 control samples. These data all come from humans. In order to expand the sample size, the “comBat” function in the SVA package is used to merge the GSE59746 and GSE75152 datasets, eliminating batch processing effects between datasets. The single-cell dataset GSE173252 contains a total of 9 samples, including 3 DD samples, 3 non pathogenic fascia samples, and 3 healthy skin samples. We divided pathogenic fascia samples and healthy skin samples into control groups, and DD samples into disease groups.

**TABLE 1:** GEO microarray chip information.

Project	GSE59746	GSE75152
Platform	GPL570	GPL6884
Species	*Homo sapiens*	*Homo sapiens*
Samples in DD group	2	12
Samples in control group	4	12
Reference	PMID: 25379672	PMID: 27467239

To expand the sample size and construct a unified combined dataset, the ComBat method in R-package SVA is used to perform batch effect correction on the datasets that need to be merged (Leek et al., 2012). This method has been widely used in genomics data analysis, especially for large gene expression datasets, by estimating and correcting unknown batch effects. Subsequently, the R package limma was used to standardize the combined dataset, including probe annotation and normalization.

### Analysis of differentially expressed genes (DEGs)

Perform differential gene expression analysis between DD samples and control samples using the “limma” package in R software, with the following criteria: |log2 (Fold Change)| > 1, adjPvalue < 0.05. The results of the differential analysis are visualized using heatmaps and volcano maps. Heatmaps are generated using the “pheatmap” package, while volcano maps are generated using the “ggplot2″ package.

### Weighted gene co expression network analysis (WGCNA) WGCNA analysis

Constructing a weighted gene co expression network using the “WGCNA” package: First, cluster the samples and remove obvious outliers. Then, a weighted adjacency matrix is constructed using a power function (adjacency function), with a focus on determining the parameter β of the adjacency function based on the scale-free network principle. Next, the topological overlap matrix (TOM) is used to calculate the degree of association between genes, and based on the TOM value, a hierarchical clustering method is used to create a clustering tree structure. Subsequently, calculate the module feature values and visualize their association with the sample shape. Finally, select the central module and obtain the core gene set corresponding to the central module. The candidate genes are the intersection of the differentially expressed gene set and the core gene set, visualized using a Venn diagram.

### Construction of protein-protein interaction (PPI) network

Import candidate genes into the STRING database, construct protein-protein interaction networks, and further use the CytoHubba plugin in Cytoscape software to calculate Node Scores, as well as the MCODE plugin to identify the most critical modules in the PPI network (Doncheva et al., 2019).

### Functional enrichment analysis

Use the “clusterProfiler” package in R software to perform Gene Ontology (GO) functional annotation and Kyoto Encyclopedia of Genes and Genomes (KEGG) pathway enrichment analysis on genes in the sub network of PPI network. A P-value less than 0.05 is considered statistically significant.

### Acquisition and evaluation of candidate biomarkers

To further screen candidate diagnostic genes, this study used genes in the sub network as candidate features and employed four machine learning algorithms for feature screening, including Least Absolute Shrinkage and Selection Operator (LASSO), Boruta, Random Forest (RF), and Support Vector Machine Recursive Feature Elimination (SVM-RFE). The LASSO algorithm reduces the risk of model overfitting to some extent by introducing L1 regularization to achieve variable selection and model simplification (Friedman et al., 2010). The Boruta algorithm is a feature selection method based on random forests, which iteratively identifies variables significantly correlated with the outcome by constructing randomly permuted shadow features and comparing the importance of real features with them (Liu et al., 2025). The RF algorithm is an ensemble supervised learning algorithm that improves the stability and accuracy of classification prediction by constructing and aggregating multiple decision trees (Tan et al., 2025). The SVM-RFE algorithm is an embedded feature selection method based on support vector machines, which recursively removes features with lower contributions and selects candidate genes with higher discriminative ability (Min et al., 2026). Subsequently, the intersection of the genes selected by the four algorithms is taken to obtain key candidate diagnostic genes with high consistency. Further combining differential expression analysis and receiver operating characteristic (ROC) curve analysis, evaluate the differential expression of these candidate genes in Dupuytran disease (DD) and their potential diagnostic value.

### Immune cell infiltration analysis

Immune infiltration analysis can clarify the composition of immune cells within the human microenvironment, which is of great significance for the study of disease pathogenesis and the proposal of treatment strategies. We used the CIBERSORT algorithm to evaluate the abundance of immune cells from the DD research cohort. The CIBERSORT algorithm provides expression data for 22 common immune infiltrating cells and can use the principle of linear vector regression to deconvolve the expression matrix of immune cell subtypes, estimate the abundance of immune cells, and draw a stacked bar chart. Subsequently, this study further investigated the differences in immune cell infiltration between the DD group and the control group, and analyzed the correlation between shared key genes and immune cells.

### Single-cell RNA-seq data processing and cell annotation

ScRNA-seq technology can help us classify clusters of sample cells. We used the “Seurat” R package to identify and process samples in the GSE173252 dataset. Firstly, create a Seurat object and perform quality control and screening on the cells to identify and remove low-quality cells. Subsequently, the remaining data was subjected to data standardization and depth scaling, and the “FindVariable Features” function was used to locate 2000 high edge genes for subsequent analysis. Principal Component Analysis (PCA) and UMAP techniques are used for dimensionality reduction and visualization of data. Next, select the appropriate resolution and use the “FindNeighbors” and “FindClusters” functions to cluster cells, generating a total of 28 cell subpopulations that were annotated as 9 immune cell subpopulations. In addition, scTenifoldKnk was used to simulate gene knockout of core targets to investigate their regulatory effects on downstream gene networks (Osorio et al., 2022).

### Pseudo time trajectory analysis

Use the Monocle software package to infer the phylogenetic trajectories of different cell subpopulations (Cao et al., 2023). Firstly, highly variable genes or differentially expressed genes are selected as sorting genes for trajectory construction, and CellDataset objects are constructed. Subsequently, the DDRTree algorithm combined with the reduceDimension function was used to reduce the dimensionality of the cells, and the orderCells function was used to sort the cells along a pseudo time trajectory to infer their dynamic developmental process.

### Construction of co expression network for key downstream genes

This study constructed a co expression network of key downstream genes using the GeneMANIA database and employed enrichment analysis to explore the potential mechanisms of action of these genes.

### Cell culture and drug therapy

Commercialized human fibroblasts are used as an inducible *in vitro* model for fibroblast activation. According to the project manuscript records, the Hs27 human fibroblast cell line (ATCC CRL-1634) was used; If different commercial fibroblast products are used in wet laboratory experiments, the cell source should be replaced accordingly. Maintain the cells in a complete culture medium recommended by the supplier at 37 °C, in a humidified atmosphere containing 5% carbon dioxide, and use within a predefined low channel range.

To promote fibrosis induction, fibroblasts were stimulated with recombinant human TGF - β 1 (5 ng/mL) for 48 h. The preliminary validation experiment compared cells treated with the carrier, cells stimulated with TGF - β 1, and cells treated with TGF - β 1 plus SB431542 (TGF - β type I receptor/ALK5 inhibitor). The numerical validation dataset corresponds to a working concentration of 10 μ M SB431542 in the three arm comparison. Under constant TGF - β 1 stimulation, parallel concentration response experiments were conducted using SB431542 at 0.1, 1, 5, and 10 mM for 48 h.

### RT-qPCR analysis

Separate total RNA after processing and reverse transcribe it into complementary DNA according to the manufacturer’s protocol. In the three arm validation experiment, RT-qPCR was used to quantify PLOD1, COL1A1, ACTA2, COL3A1, SERPINE1, FN1, and CCN2. PLOD1 was selected as the priority target in the computational workflow, while ACTA2, COL1A1, COL3A1, FN1, CCN2, and SERPINE1 were used as markers for myofibroblast activation, extracellular matrix production, and TGF - β - reactive fibrosis signaling. For the SB431542 concentration reaction experiment, PLOD1, COL1A1, and ACTA2 were measured as the core validation endpoints. RPLP0 is used as an internal reference gene in the project calculation table. Calculate the relative mRNA abundance using the 2^-△△CT^ method. The target gene sequence is shown in Table 2.

**TABLE 2:** The target gene sequence.

Full name of gene	Gene	Forward primer (5’→3′)	Reverse primer (5’→3′)
Procollagen-lysine, 2-oxoglutarate 5-dioxygenase 1	PLOD1	GCTGCTGCTGTTCTACCTGA	CAGGGTGATGTTGGTGATGG
Collagen type I alpha 1	COL1A1	GAGGGCCAAGACGAAGACATC	CAGATCACGTCATCGCACAAC
Collagen type III alpha 1 chain	COL3A1	TGGTCCTGCTGGAAAGGATG	CCTGGTTGCTGGTGTTTTGG
Actin alpha 2	ACTA2	GTGTTGCCCCTGAAGAGCAT	GCTGGGACATTGAAAGTCTCA
Fibronectin 1	FN1	ATGTGGACCCCTCCTGATAGT	GCCCAGTGATTTCAGCAAAGG
Cellular communication network factor 2	CCN2	CCAATGACAACGCCTCCTG	TGGTGCAGCCAGAAAGCTC
Serpin family E member 1	SERPINE1	AGTGGAGAGTCTTCAGTGCCT	CTTTGTGTCCAGGAGGTGCT
Ribosomal protein lateral stalk subunit P0	RPLP0	GCAATGTTGCCAGTGTCTGT	GCCTTGACCTTTTCAGCAAG

### CCK-8 activity assay

After 48 h of processing, the cell survival rate/metabolic activity was measured using the CCK-8 assay method. Add CCK-8 reagent to each well, incubate according to the manufacturer’s instructions, and then measure absorbance at the recommended wavelength. The value is expressed as a percentage of survival rate relative to the corresponding reference conditions. The experiment aims to evaluate whether transcriptional repression of fibrosis markers occurs within a concentration range that preserves overall cellular metabolic activity.

### Statistical analysis

R software (version 4.3.0) is used for data processing and analysis. The data is presented as the average ± SEM of six repeated observations plotted for each group. For multi group comparisons, use one-way ANOVA and then perform the pre specified pairwise comparisons shown in the figure. For the dose-response group, as shown in parentheses, a comparison was made between adjacent SB431542 dose groups. A bilateral P value < 0.05 is considered statistically significant. The graphic symbols are defined as *P < 0.05, * *P < 0.01, * *P < 0.001, and ns, which are not significant. Before submission, the final manuscript should verify whether the drawn observations represent independent biological replication, independent experiments, or technical replication.

## Result

### Screening of key genes between DD and control group

Firstly, this study identified 598 DEGs between the DD group and the control group (Figures 1A,B). Then, to identify the core genes associated with DD, we constructed a gene co expression network using WGCNA. Sample clustering analysis reveals clear clustering of samples, indicating reliable data quality. By selecting a soft threshold of β = 4, a scale-free topological fitting index (R^2^) of 0.85 was obtained, which confirms the construction of the scale-free co expression network (Figure 1C). Using a gene correlation matrix, we constructed a hierarchical clustering tree diagram of genes and identified 5 different gene modules (Figure 1D). Among them, the MEbrown module containing 806 genes showed the strongest correlation with the DD phenotype (Cor = 0.83, p = 1e-08) (Figure 1E). The scatter plot shows a significant positive correlation (Cor = 0.81, p = 1.5e-188) between gene significance (GS) within the “brown” module and module members (MM), indicating a high degree of consistency between module genes and DD correlation (Figure 1F).

**FIGURE 1:**
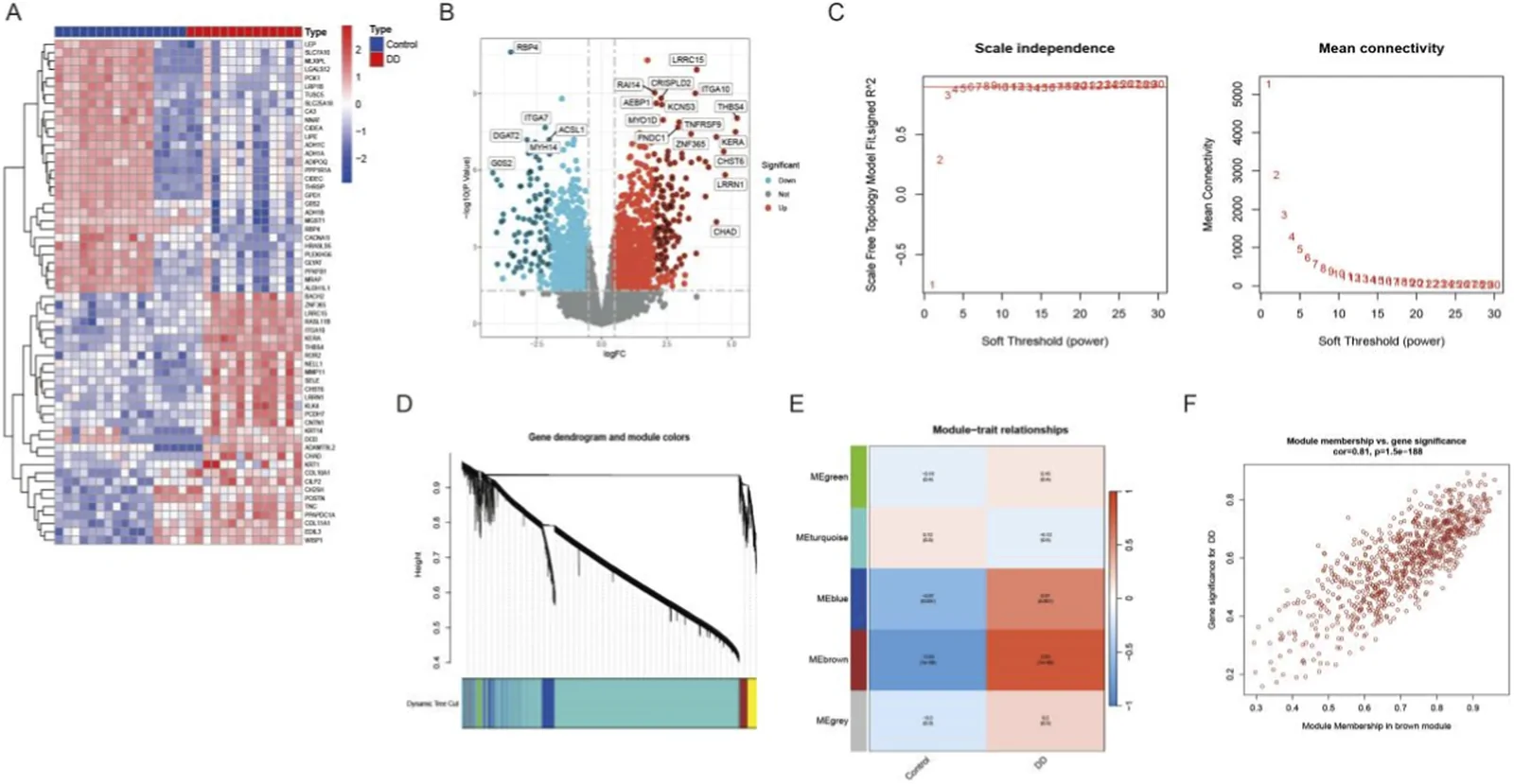
Screening of key genes. **(A)** Heat map of DEGs. **(B)** The volcano map of DEGs. **(C)** Soft threshold determination. **(D)** Hierarchical clustering tree diagram of gene co expression modules. **(E)** Module shape relationship heatmap. **(F)** Scatter plot of MM and DD genes GS in the brown module.

### PPI network analysis

This study intersected the core genes contained in the brown modules of DEGs and WGCNA to obtain 346 candidate genes contributing (Figure 2A). To further investigate the function of candidate genes, we constructed a PPI network (Figure 2B), which includes 343 nodes and 729 edges. Then, we obtained the key subnetworks through the cytoHubba plugin and MCODE app. In order to explore the potential mechanisms of key genes in the key sub network in DD, we performed functional annotation analysis on these genes (Figure 2C). The results of GO enrichment analysis showed that in terms of BP, genes were mainly enriched in external wrapped structural tissues, extracellular structural tissues, and extracellular matrix tissues. In terms of CC, they mainly focus on the extracellular matrix, collagen trimer, and endoplasmic reticulum lumen contained in collagen. In terms of MF, it mainly participates in molecular functions such as extracellular matrix structural components (Figure 2D). The results of KEGG analysis show that these candidate genes are mainly enriched in protein digestion and absorption (Figure 2E).

**FIGURE 2:**
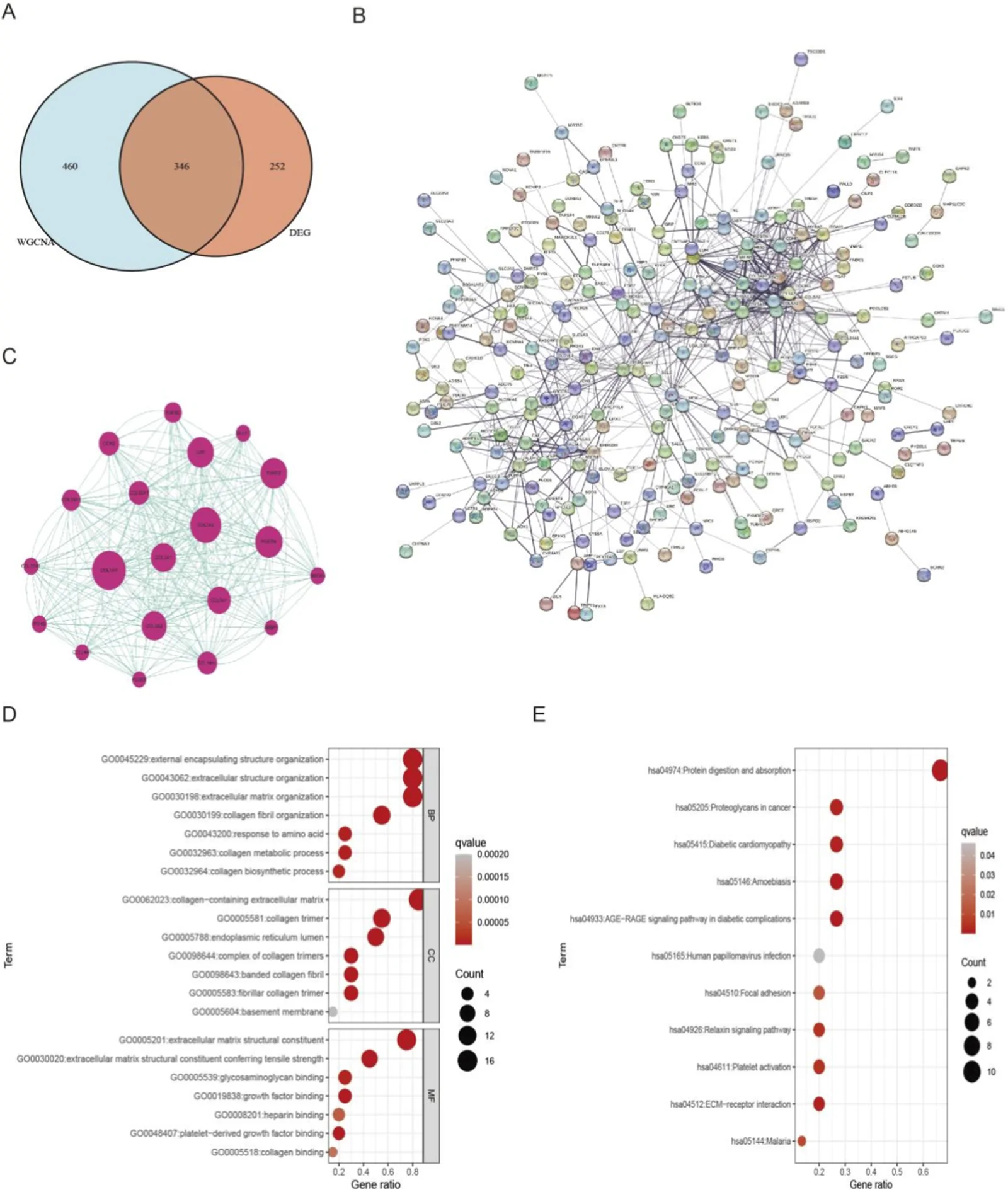
PPI network analysis and functional annotation. **(A)** The Venn diagram displays the intersection genes of DEG and WGCNA core genes. **(B)** Import candidate genes into the STRING database to construct a PPI network. **(C)** PPI network imports Cytoscape, CytoHubba plugin, and MCODE app to obtain key subnetworks. **(D)** Perform GO analysis on the genes of the sub network. **(E)** Perform KEGG analysis on the genes of the sub network.

### Identification of key biomarkers

Screen key candidate genes in key sub networks using four different machine learning algorithms. The LASSO regression algorithm identified 6 genes (Figures 3A,B), the Boruta algorithm identified 13 genes (Figures 3C,D), the random forest algorithm identified 6 genes (Figures 3E,F), and the svm ref algorithm identified 11 genes (Figures 3G,H). Perform intersection analysis on the results obtained from screening four machine learning algorithms to obtain a shared candidate biomarker (PLOD1) (Figure 3I). The expression of PLOD1 in the DD group was significantly higher than that in normal tissues (P = 0.00012) (Figure 3J). To verify the diagnostic potential of the biomarker PLOD1, we applied ROC curve analysis with an area under the curve (AUC) of 0.908, indicating that PLOD1 has excellent clinical diagnostic value for DD (Figure 3K).

**FIGURE 3:**
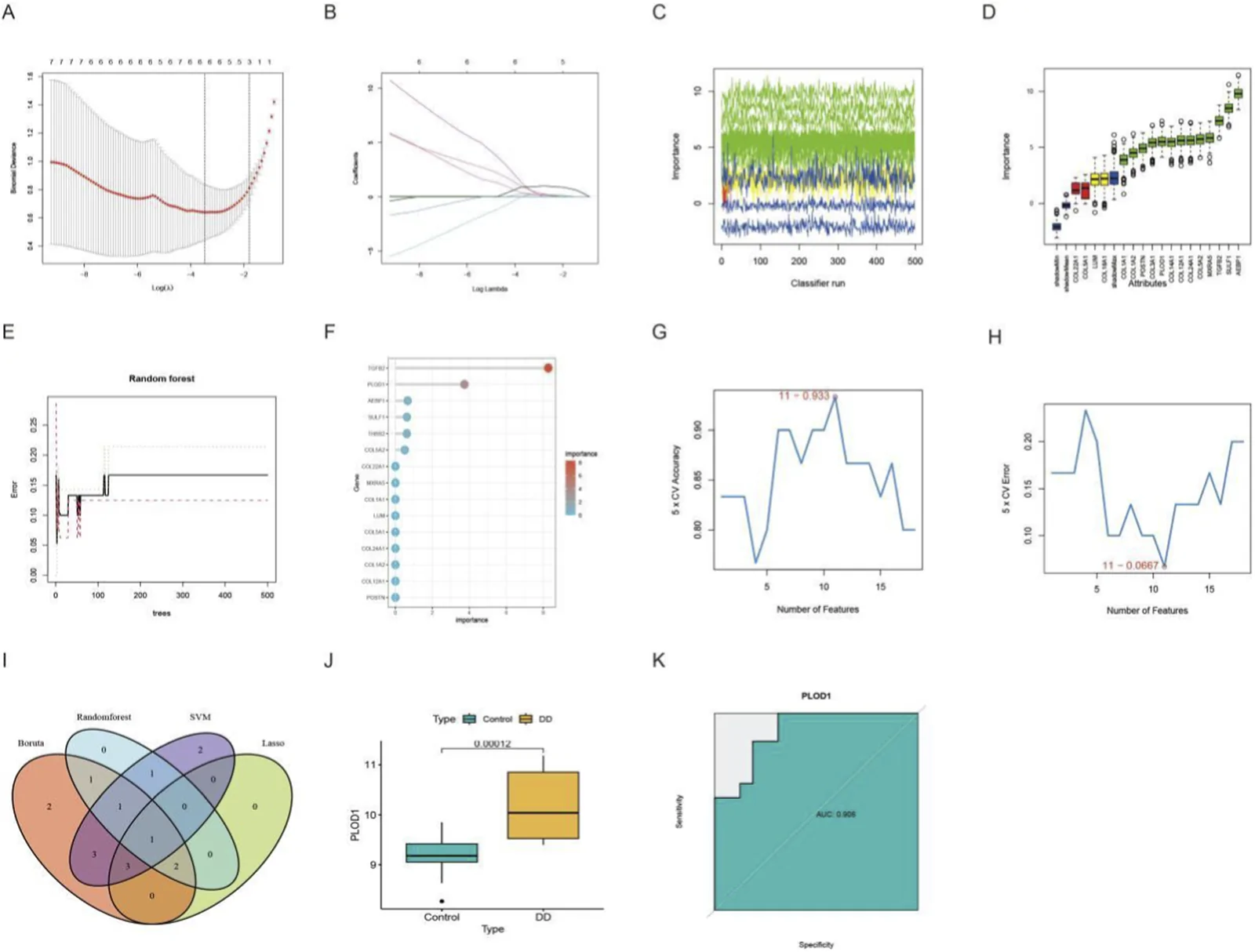
Screening and identification of biomarkers. **(A,B)** LASSO algorithm. **(C,D)** Boruta algorithm. **(E,F)** Random Forest Algorithm. **(G,H)** SVM ref algorithm. **(I)** The Venn diagram shows the intersection genes of feature genes obtained by four machine learning algorithms. **(J)** The expression differences of shared candidate biomarkers between DD and normal groups. **(K)** ROC curves of shared candidate biomarkers.

### Immune cell infiltration analysis

The CIBERSORT algorithm was used to evaluate the abundance of immune cells in 30 samples from the DD research cohort, with the proportion of each immune cell shown in Figure 4A. Compared with the control group, the infiltration level of Macrophages M0 in the DD group significantly decreased (Figure 4B). The biomarker PLOD1 was significantly positively correlated with M2 macrophages (P = 0.041). (Figure 4C).

**FIGURE 4:**
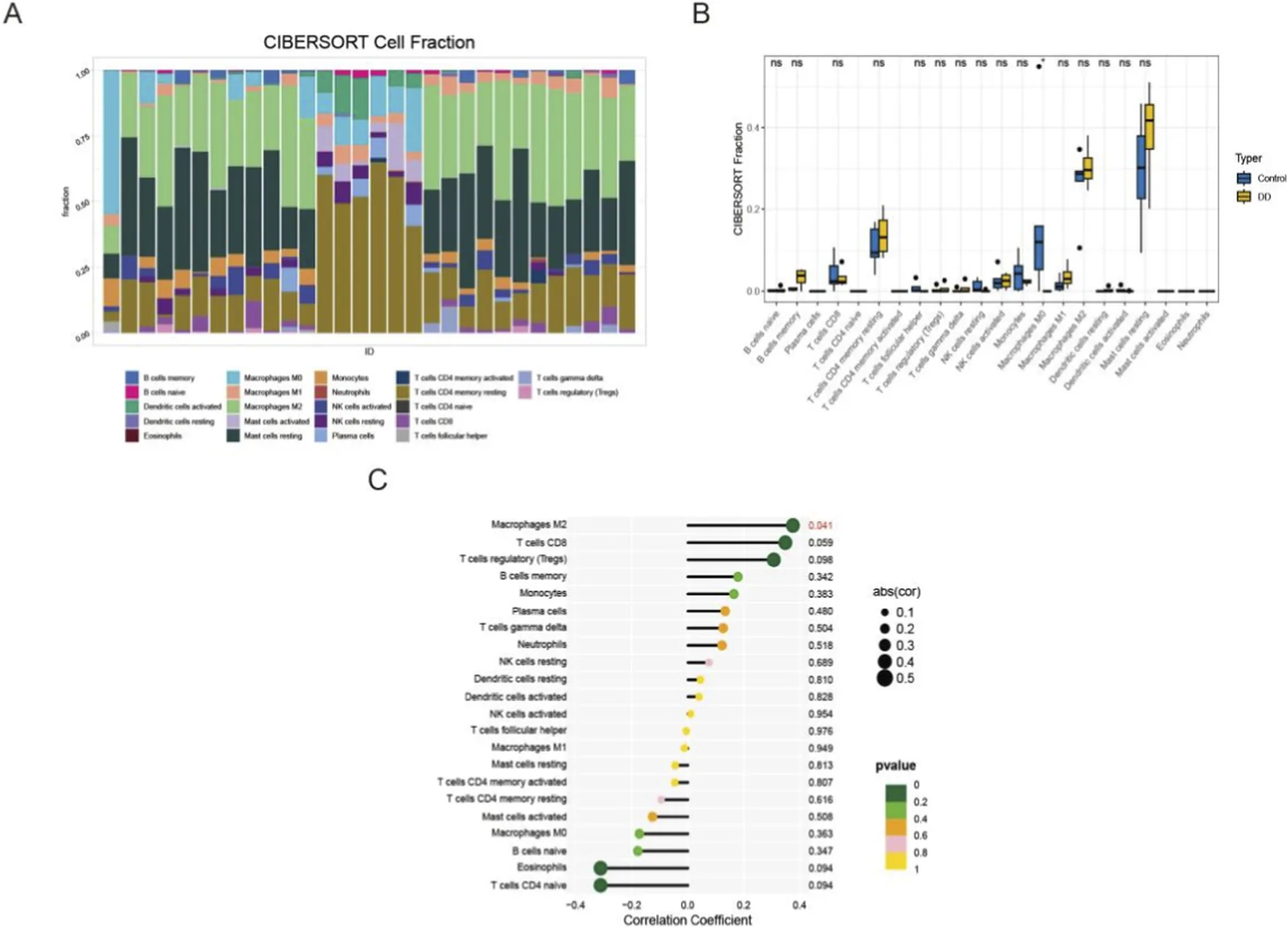
Analysis of immune cell infiltration. **(A)** The proportion of immune cell infiltration in each sample. **(B)** The difference in immune cell infiltration levels between the control group and the DD group. **(C)** PLOD1 is significantly correlated with various immune cells.

### The dynamic characteristics of PLOD1 expression in the development trajectory of DD

In single-cell data analysis, we deconstructed the cellular landscape of two clinical cohorts, the control group and DD. By using typical biomarkers to divide cell populations, 9 cell subgroups were identified, including chondrocytes, fibroblasts, B cells, macrophages, tissue stem cells, epithelial cells, T cells, endothelial cells, and monocytes (Figure 5A). The expression of PLOD1 is mainly associated with chondrocyte related cell populations (Figure 5B).

**FIGURE 5:**
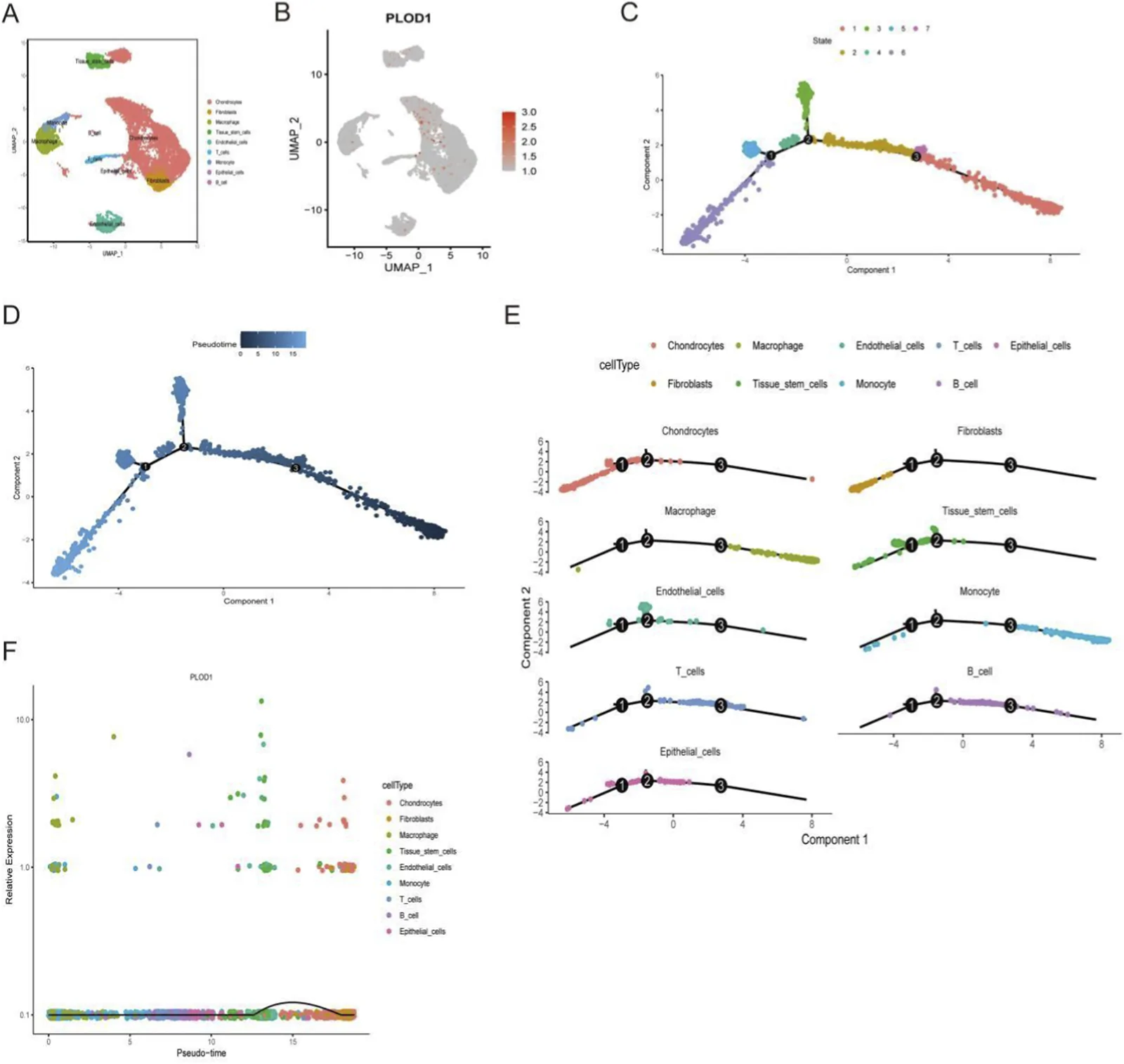
Dynamic characteristics of PLOD1 expression in the development trajectory of DD. **(A)** UMAP visualization of single-cell data. **(B)** UMAP plot of PLOD1 expression. **(C)** The cell trajectory state diagram displays the developmental trajectories of cells at different pseudo time points, with 6 cell states displayed in different colors, and all subgroups displayed in the trajectory. **(D)** The pseudo time chart simulates the sequence of cell differentiation. **(E)** The cell type map shows that each cell cluster is on a different differentiation trajectory. **(F)** The relative expression of PLOD1 at different pseudo times.

In order to simulate the dynamic gene expression during the continuous development of DD, Monocle 2 conducted trajectory analysis based on transcriptional changes during cell differentiation to reconstruct the developmental trajectory of cells. We found that branch points 1–3 divide cell trajectories into six future cell states. The direction of cell trajectory is from right to left (Figures 5C,D). Chondrocytes are in the late stage of differentiation (Figure 5E). In addition, PLOD1 was found to be upregulated in the late stage trajectory, most commonly in local clusters of chondrocytes (Figure 5F).

### Knockout of PLOD1 can regulate downstream networks

In order to investigate the molecular regulatory function of PLOD1 gene, this study conducted virtual knockout analysis on PLOD1. The results showed that after knocking out PLOD1, FGF7 was the most severely interfered downstream gene (Figures 6A,B). Then, this study constructed a co expression network of FGF7 using the geneMANIA database (Figure 6C). To explore the potential mechanisms of action in these genes, we conducted functional enrichment analysis. The results of KEGG analysis show that these genes are associated with PI3K-Akt signaling pathway, MAPK signaling pathway,Ras signaling pathway, Regulation of actin cytoskeleton The pathways are significantly correlated (Figure 7D). The results of GO analysis show that these genes are associated with fibroblast growth factor receptor signaling pathway, cellular response to fibroblast growth factor stimulus, response to fibroblast growth factor, fibroblast growth factor receptor binding The pathways are significantly correlated (Figure 6E).

**FIGURE 6:**
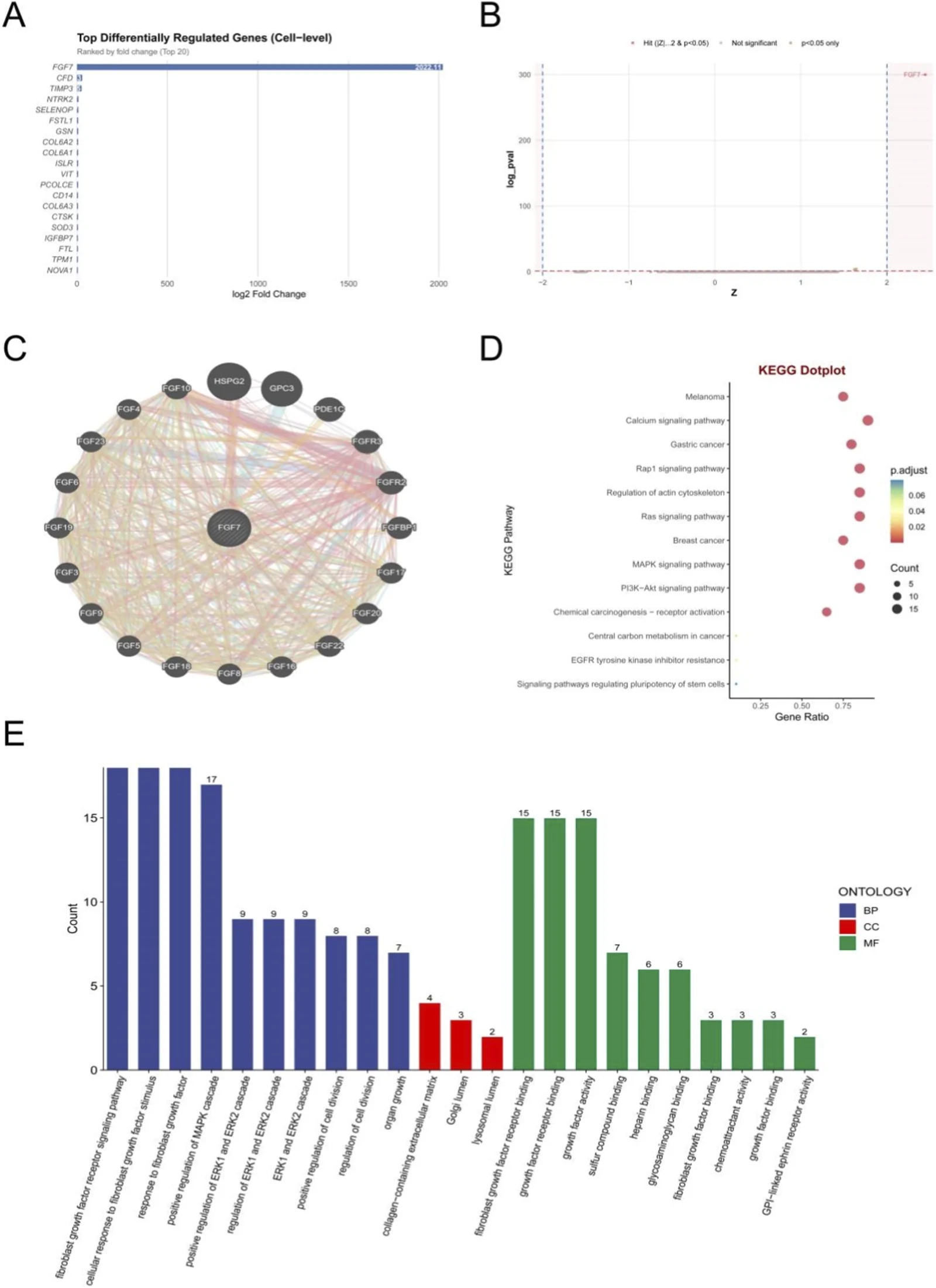
Knockout of PLOD1 identifies downstream regulatory networks. **(A,B)** The result of PLOD1 virtual knockout. **(C)** The co expression network of FGF7. **(D)** KEGG analysis of co expressed network genes. **(E)** GO analysis of co expressed network genes.

**FIGURE 7:**
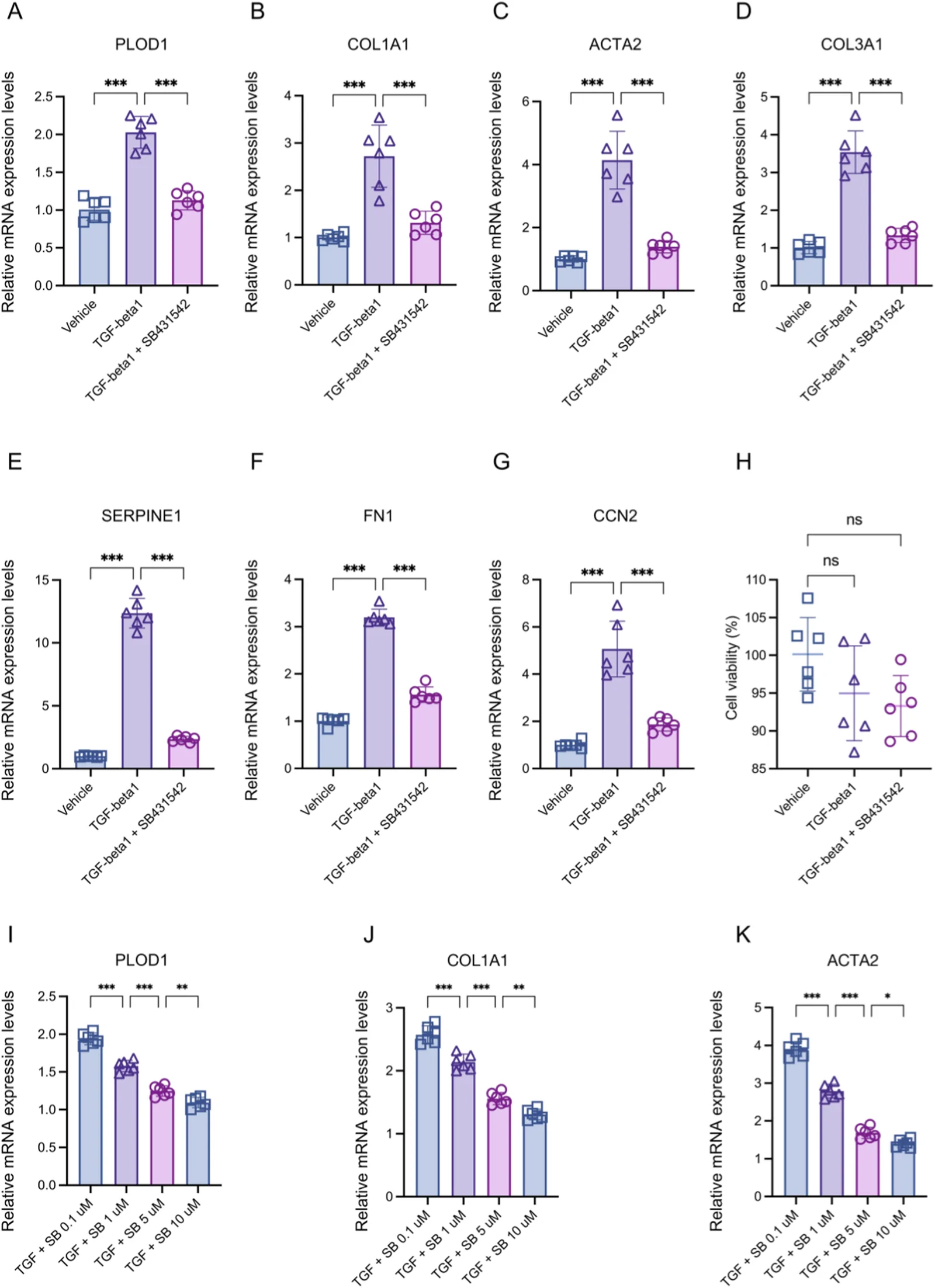
SB431542-sensitive regulation of the PLOD1-associated fibrotic transcriptional program in TGF-beta1-stimulated human fibroblasts. Commercial human fibroblasts were treated with vehicle, TGF-beta1, or TGF-beta1 plus SB431542 for 48 h. Relative mRNA expression was measured by RT-qPCR and calculated using the 2^−△△CT^ method with RPLP0 as the internal reference gene. **(A)** PLOD1, **(B)** COL1A1, **(C)** ACTA2, **(D)** COL3A1, **(E)** SERPINE1, **(F)** FN1, and **(G)** CCN2 mRNA expression in the three-arm validation experiment. **(H)** Cell viability/metabolic activity measured by CCK-8 assay in the same treatment groups. For dose-response validation, fibroblasts were maintained under TGF-beta1 stimulation and co-treated with SB431542 at 0.1, 1, 5, or 10 microM for 48 h; **(I)** PLOD1, **(J)** COL1A1, and **(K)** ACTA2 mRNA expression are shown. Data are presented as mean±SEM; n = 6 plotted replicate observations per group. Statistical comparisons were performed by one-way ANOVA followed by the prespecified pairwise comparisons indicated by brackets. *P < 0.05, **P < 0.01, ***P < 0.001; ns, not significant.

### TGF-β1 induces PLOD1 expression and a profibrotic transcriptional program

Based on the predominant localization of PLOD1 in the chondrocyte population identified by single-cell analysis, we further examined its regulation in an *in vitro* model of fibrotic activation. RT-qPCR analysis showed that TGF-β1 treatment significantly increased PLOD1 mRNA expression compared with the vehicle control (Figure 7A). Consistent with the induction of a profibrotic phenotype, TGF-β1 also markedly upregulated the extracellular matrix- and fibrosis-associated genes COL1A1, ACTA2, COL3A1, SERPINE1, FN1, and CCN2 (Figures 7B–G). Co-treatment with the TGF-β signaling inhibitor SB431542 significantly attenuated the TGF-β1-induced increases in PLOD1 and all examined profibrotic markers, indicating that their transcriptional activation was dependent on TGF-β signaling. Cell viability did not differ significantly among the vehicle, TGF-β1, and TGF-β1 plus SB431542 groups (Figure 7H), suggesting that the inhibitory effects of SB431542 on gene expression were not attributable to overt cytotoxicity. Furthermore, under continuous TGF-β1 stimulation, increasing the concentration of SB431542 from 0.1 to 10 μM progressively reduced the mRNA levels of PLOD1, COL1A1, and ACTA2 (Figures 7I–K), demonstrating a concentration-dependent inhibitory effect. Collectively, these findings identify PLOD1 as a TGF-β1-responsive component of the profibrotic transcriptional program and show that pharmacological blockade of TGF-β signaling suppresses both PLOD1 expression and associated extracellular matrix and myofibroblast activation markers in DD.

## Discussion

DD, Palm fascia fibrosis, as a fibroproliferative disease, can cause serious functional damage and ultimately lead to hand disability in patients (Hindocha et al., 2008). Although there are currently multiple non-surgical treatment methods, their effectiveness is limited (Ketchum and Donahue, 2000; Pittet et al., 1994; Richards, 1952; Vuopala and Kaipainen, 1971). Moreover, surgical removal of contracted tissue carries significant risks, including damage to the finger nerves and blood vessels, injury to the bent tendons, and failure of wound healing that may lead to skin necrosis. Previous studies have revealed the involvement of multiple genes in the pathogenesis of DD, but reliable molecular biomarkers and regulatory factors have not yet been fully determined. In this study, we integrated batch transcriptome analysis, weighted gene co expression network analysis, protein interaction analysis, multiple machine learning algorithms, immune infiltration estimation, single-cell transcriptome analysis, virtual gene knockout prediction, and *in vitro* validation. Through this multi-level strategy, we identified PLOD1 as a key diagnostic and mechanistic biomarker associated with DD.

By analyzing the intersection of genes in the DEGs and DD related WGCNA modules, we obtained 346 candidate genes and screened the key sub networks of PPI. The functional enrichment analysis of key subnetworks indicates that these genes are mainly involved in extracellular matrix tissue, extracellular structural components, collagen containing extracellular matrix, collagen trimer formation, and protein digestion and absorption pathways. These findings are highly consistent with the known pathological features of DD. The main feature of DD is the accumulation of extracellular matrix proteins, forming abnormal connective tissue in the palmar fascia, mainly containing collagen (especially type I and III) and myofibroblasts (Bazin et al., 1980). Therefore, the enrichment of extracellular matrix and collagen related biological processes supports the effectiveness of our bioinformatics screening strategy.

Using four independent machine learning algorithms, including LASSO regression Boruta, Random forest and SVM-RFE, we identified PLOD1 as the only overlapping candidate biomarker. The convergence of multiple algorithms enhances the robustness of this discovery and reduces algorithm specific biases. Compared with the normal control group, the expression of PLOD1 in DD tissues significantly increased, and ROC curve analysis showed an AUC of 0.908, indicating excellent diagnostic performance. Pre collagen lysyl hydroxylase (PLOD) is essential for regulating collagen synthesis and cross-linking, promoting the maturation of extracellular matrix. Procollagen-lysine, 2-oxoglutarate 5-dioxygenase 1 (PLOD1) is a member of the PLOD protein family. PLOD1 is important for the stability of intermolecular deposition and cross-linking (Chen and Wu, 2025). The core pathological process of DD is abnormal fibrosis. The occurrence and progression of DD are mainly manifested by abnormal activation of fibroblasts in the palmar fascia, which further differentiate into myofibroblasts. These activated cells can synthesize and deposit a large amount of extracellular matrix (ECM) components, including type I and III collagen, fibronectin, proteoglycans, and periostin, leading to gradual thickening and hardening of local tissues, and ultimately forming persistent fibrous scar like structures (Wynn and Ramalingam, 2012; O’Gorman et al., 2017; Ehrlich et al., 1982; Williamson et al., 2025). In the early stages of the disease, fibrous nodules with rich cellular components often appear in the palmar fascia, which are considered to represent the “active phase” of DD. The enriched myofibroblasts in nodules are key effector cells driving local fibrosis responses. As the disease progresses, abnormally deposited collagen fibers gradually arrange and reshape along the direction of the palmar fascia, forming typical fibrous strands. Myofibroblasts not only continuously secrete ECM, but also have strong contraction ability. They can apply tension to ECM through local adhesive structures, thereby pulling fingers and ultimately leading to progressive flexion contracture (O’Gorman et al., 2017; Khaliq and Orji, 2024; Nanchahal and Chan, 2023; Verjee et al., 2009). From the perspective of cell phenotype, myofibroblasts typically express α-smooth muscle actin (α-SMA) and form stress fibers, which are an important basis for generating mechanical contraction force. At the same time, myofibroblasts also showed enhanced ECM protein synthesis ability, especially increased production of type I and III collagen (Puerta Cavanzo et al., 2022). Therefore, DD can be regarded as a locally progressive fibrotic disease driven by abnormal collagen deposition, ECM remodeling, and myofibroblast mediated mechanical contraction. Based on this pathological feature, intervening in the fibrosis process may help delay or improve the disease progression of DD. In recent years, research on fibrosis related molecular targets has provided new ideas for the treatment of DD. Miao Xu et al. found that direct or indirect inhibition of PLOD1 can significantly enhance the anti fibrotic potential of adipose derived mesenchymal stem cells (AMSCs) during wound healing by regulating macrophage polarization (Xu et al., 2021). Given that PLOD1 is closely related to collagen maturation, cross-linking, and fibrosis processes, targeting PLOD1 may help inhibit abnormal ECM deposition and tissue fibrosis, providing potential therapeutic strategies for improving the occurrence and development of DD.

Although DD has long been regarded as a disease characterized by excessive proliferation of fibroblasts and abnormal ECM remodeling, increasing evidence suggests that immune and inflammatory processes play a crucial role in initiating and maintaining palmar fascia fibrosis. The immune infiltration analysis in this study further indicates that the composition of immune cells in DD tissues has changed. It is worth noting that compared with the control group, the infiltration of M0 macrophages in the DD group was significantly reduced, while the expression of PLOD1 was positively correlated with M2 macrophages. M2 macrophages have been shown to be an important source of pro fibrotic cytokines (Izadi et al., 2019). The positive correlation between PLOD1 and M2 macrophages suggests that PLOD1 related matrix remodeling may occur in the pro fibrotic immune microenvironment. This discovery increases the possibility that the interaction between macrophage polarization and collagen processing enzymes may be involved in the progression of DD. However, these findings are inferred from bulk transcriptome and may be influenced by differences in tissue cell count, stromal cell content, and extracellular matrix composition. Therefore, the observed association should not be interpreted as evidence that PLOD1 regulates M2 macrophages or the immune microenvironment. The exact causal relationship between PLOD1 expression and macrophage infiltration needs further experimental verification.

Single cell transcriptome analysis provides additional insights into the cellular origin and dynamic expression patterns of PLOD1 in DD. Nine main cell populations were identified, including chondrocytes, fibroblasts, macrophages, T cells, B cells, endothelial cells, epithelial cells, monocytes, and tissue stem cells. In these populations, the expression of PLOD1 is mainly associated with chondrocyte related cell populations. Pseudo time trajectory analysis further indicates that chondrocytes are in the late differentiation stage, and PLOD1 expression increases along the late developmental trajectory. These results suggest that PLOD1 may be involved in terminal or activated cell states associated with DD progression. Although DD has traditionally been considered a fibroproliferative disease, increasing evidence suggests that stromal cells associated with the disease may exhibit heterogeneous transcriptional phenotypes, including chondrocyte like or stromal producing cell states. Therefore, the enrichment of PLOD1 in chondrocyte associated clusters may reflect a specific extracellular matrix production population involved in collagen remodeling and disease progression.

In order to further explore the regulatory role of PLOD1, virtual knockout analysis was conducted. The results indicate that FGF7 is the most severely affected downstream gene after PLOD1 knockout. The fibroblast growth factor (FGF) family plays a crucial role in various cellular processes, including cell proliferation, differentiation, migration, and survival (Beenken and Mohammadi, 2009). Among them, FGF7 is mainly expressed in epithelial tissues such as skin, lungs, gastrointestinal tract, and kidneys (Finch et al., 1989). Previous studies have shown that in CCL4 induced acute liver injury models, FGF7 has been shown to contribute to liver cell survival, thereby reducing the degree of fibrosis (Geervliet et al., 2023). In addition, FGF7 can guide stem cells to differentiate into the tendon lineage while inhibiting fibrosis differentiation within the tendon. Further research suggests that FGF7 is an important regulatory factor that inhibits fibrosis and promotes connective tissue morphogenesis (Lin et al., 2025). Therefore, the PLOD1-FGF7 regulatory axis may represent a potential mechanism that links collagen processing and extracellular matrix remodeling with tissue repair, cell differentiation, and fibrosis regulation processes.

Importantly, we further validated the regulatory relationship between *in vitro* pro fibrotic stimulation and PLOD1 expression. TGF - β 1 treatment significantly induces PLOD1 mRNA expression, while upregulating classical extracellular matrix and fibrosis related genes, including COL1A1, COL3A1, ACTA2, SERPINE1, FN1, and CCN2. These genes are central mediators of myofibroblast activation and fibrotic extracellular matrix deposition. The TGF - β receptor inhibitor SB431542 significantly reduced the expression of PLOD1 induced by TGF - β 1, and all detected pro fibrotic markers did not affect cell survival, indicating that these effects were not due to cytotoxicity. In addition, under continuous TGF - β 1 stimulation, the increasing concentration of SB431542 gradually inhibited the expression of PLOD1, COL1A1, and ACTA2, confirming the concentration dependent inhibitory effect. These results indicate that PLOD1 is a TGF - β 1 responsive gene and that PLOD1 is involved in the TGF - β - driven pro fibrotic transcription program in DD.

However, some limitations should be acknowledged. Firstly, the sample size of public transcriptome datasets is relatively limited, requiring a larger independent queue to validate the diagnostic performance of PLOD1. Secondly, although ROC analysis indicates strong diagnostic value, it is necessary to conduct prospective clinical studies to determine whether PLOD1 can distinguish different stages, severity, or recurrence risk of DD. Thirdly, immune infiltration results are inferred from a large amount of transcriptome data and should be validated through immunohistochemistry, flow cytometry, or spatial transcriptomics. Fourthly, although virtual knockout analysis suggests that FGF7 is a downstream target of PLOD1, this regulatory relationship still has predictive value and requires experimental validation using PLOD1 knockout or overexpression models. Finally, although our *in vitro* data demonstrated TGF - β - dependent regulation of PLOD1, additional functional testing is needed to determine whether PLOD1 directly regulates collagen cross-linking, matrix hardness, fibroblast contractility, and myofibroblast differentiation in DD derived primary cells.

## Conclusion

In summary, this study identified PLOD1 as a robust diagnostic biomarker and potential functional regulator for DD. PLOD1 is highly expressed in DD tissues and exhibits excellent diagnostic performance. It is associated with extracellular matrix remodeling and M2 macrophage infiltration, and is mainly localized in chondrocyte related cell populations in single-cell analysis. These findings provide new insights into the molecular pathogenesis of DD.

## Data Availability

The data presented in the study are deposited in the GEO repository, accession number: GSE59746, GSE75152, and GSE173252.
